# Hybrid Total Achilles Tendon Reconstruction Combining Acellular Dermal Matrix Placement and Free Latissimus Dorsi Flap Coverage: A Report of Two Cases

**DOI:** 10.1002/micr.70146

**Published:** 2025-11-19

**Authors:** Beniamino Brunetti, Chiara Camilloni, Matteo Pazzaglia, Valeria Petrucci, Marco Morelli Coppola, Rosa Salzillo, Stefania Tenna, Irene Giovanna Aprile, Marco Germanotta, Sergio Valeri, Mauro Barone, Paolo Persichetti

**Affiliations:** ^1^ Operative Research Unit of Plastic, Reconstructive and Aesthetic Surgery Fondazione Policlinico Universitario Campus Bio‐Medico Rome Italy; ^2^ Research Unit of Plastic Surgery, Department of Medicine and Surgery Università Campus Bio‐Medico di Roma Rome Italy; ^3^ IRCCS Fondazione Don Carlo Gnocchi Florence Italy; ^4^ Operative Research Unit of Soft‐Tissue Sarcomas Surgery Department Fondazione Policlinico Universitario Campus Bio‐Medico Rome Italy

**Keywords:** Achilles tendon, free flap, microsurgery, regenerative medicine, repair

## Abstract

Reconstruction of complex Achilles tendon defects involving both the tendon and the overlying soft tissues represents a challenging scenario for plastic surgeons. In this report, we present an innovative technique of hybrid total Achilles tendon reconstruction combining acellular dermal matrix placement to restore the full‐thickness continuity of the tendon and free latissimus dorsi (LD) flap coverage to allow graft integration and simultaneously resurface the soft tissue defect, ultimately leading to lower limb salvage. Between August 2023 and February 2024 two patients received microsurgical hybrid reconstruction of complex defects, measuring 10 × 10 cm and 17 × 14 cm, respectively, involving the lower third of the leg and the Achilles tendon region, due to trauma with multiple previous failed surgeries and sarcoma resection. The full‐thickness continuity of the tendon was reconstructed by use of an XCM BIOLOGIC Tissue Matrix (DePuy Synthes, Johnson & Johnson), measuring 6 and 10 cm long, respectively, folded in a three‐layered tridimensional structure to recreate the shape and function of a new tendon. Subsequently, a free myo‐cutaneous LD flap, was used to wrap and revascularize the neo‐tendon and resurface the soft tissue defect. In both patients the flaps healed uneventfully, and neo‐tendon integration was documented by MRI examination. The follow‐up was uneventful. Long‐term functional evaluation showed almost normal tendon excursion with both patients walking without assistance. The proposed hybrid approach may be a good alternative and innovative solution for the reconstruction of complex Achilles tendon defects consequent to trauma or oncological resection. Such results are more than promising for future studies on large series of patients.

## 1 Introduction

Reconstruction of complex Achilles tendon defects involving both the tendon and the overlying soft tissues represents a challenging scenario for plastic surgeons. Pedicled flaps such as the reverse sural, the medial plantar artery (Elhefnawy et al. 2023), the posterior tibial artery, or peroneal artery perforator flaps (Jakubietz et al. 2010) represent a workhorse solution for small‐ to moderate‐size defects with minor tendon involvement, while free flaps are preferred in case of more extensive defects requiring total/subtotal tendon replacement (Rein and Kremer 2022).

Taking two birds with one stone, the ALT‐fascia lata chimeric flap has been described many times for this kind of reconstructions (Son et al. 2022; Tiengo et al. 2020), providing reliable functional results with lower donor site morbidity when compared to the composite radial forearm flap harvested with flexor carpi radialis tendon (Maffulli and Ajis 2008). Unfortunately, in major defects even this strategy might not suffice: The load an Achilles tendon has to bear is so high that not many structures in the body are equipped to withstand it, and the tendon complex structure and viscoelastic properties are impossible to replicate (Finni and Vanwanseele 2023).

To solve this problem some surgeons have proposed strengthening the reconstructed tendon with a synthetic mesh (McCartney et al. 2019) or an acellular dermal matrix (Cole et al. 2018), with good results. Even this considered, there are no descriptions in the literature, to the best of our knowledge, of a full‐thickness Achilles tendon reconstruction with an acellular dermal matrix, used alone as a neo‐tendon allograft to bridge the tendon gap and restore tendon continuity and function.

In this paper, we present an innovative technique of hybrid total Achilles tendon reconstruction combining acellular dermal matrix placement to restore the full‐thickness continuity of the tendon and free latissimus dorsi (LD) flap coverage to allow graft integration and simultaneously resurface the soft tissue defect, ultimately leading to lower limb salvage. This report represents the first description in the literature about the reconstruction of a new Achilles tendon using the combination of regenerative surgery and microsurgery.

## 2 Case Reports

### 2.1 Case 1

**FIGURE 1:**
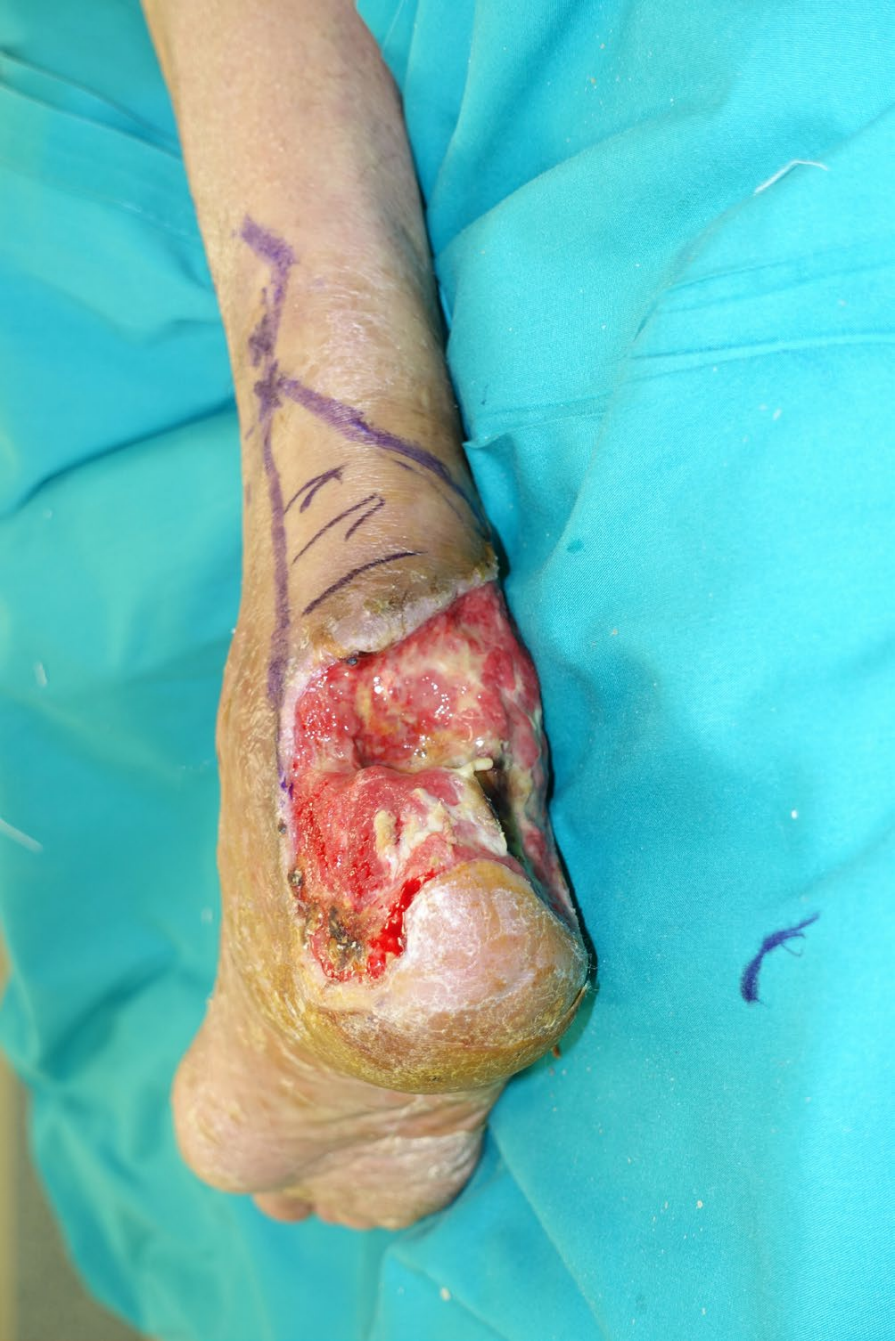
Post debridement presentation of soft tissue defect plus 4.5‐cm full‐thickness abrupt interruption of the Achilles tendon (with only the proximal stump still left in place) and calcaneal bone and distal tibia exposure.

**FIGURE 2:**
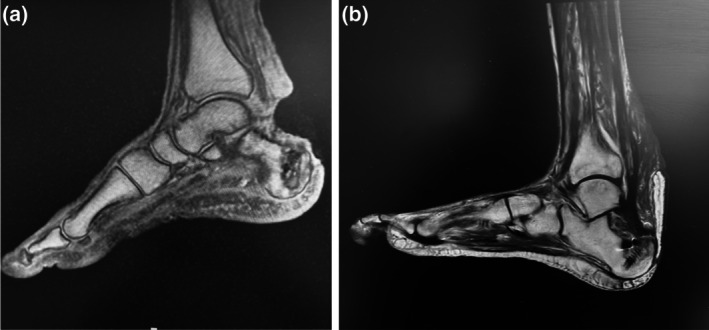
(a) Preoperative MRI showed extensive necrosis of the retro‐calcaneal area including most of the Achilles tendon, proximal displacement of the Achilles tendon proximal stump, severe erosion of the calcaneal bone, and possible FHL tendon involvement; (b) 6 months postoperatively MRI shows complete restoration of the Achilles tendon.

A 52‐year‐old male with multiple comorbidities was referred to our team to treat a 10 × 10 cm soft tissue defect that included a 4.5‐cm full‐thickness abrupt interruption of the Achilles tendon (with only the proximal stump still left in place) and calcaneal bone and distal tibia exposure (Figure 1), following unspecified tendon repair surgery at another center. Preoperative MRI showed extensive necrosis of the retro‐calcaneal area including most of the Achilles tendon (Figure 2a). One week after debridement, a planned hybrid reconstruction was performed: Tendon continuity was restored creating a 6‐cm‐long neo‐tendon by use of an XCM BIOLOGIC Tissue Matrix (DePuy Synthes, Johnson & Johnson) folded in a strong, three‐layered tridimensional structure, secured with reabsorbable sutures. The neo‐tendon was first inserted distally with anchor screws at the level of the calcaneal bone and then sutured to the Achilles tendon proximal stump or to the remnants of the calf effectors with Prolene 0 under maximal tension (dorsal flexion of the foot).

**FIGURE 3:**
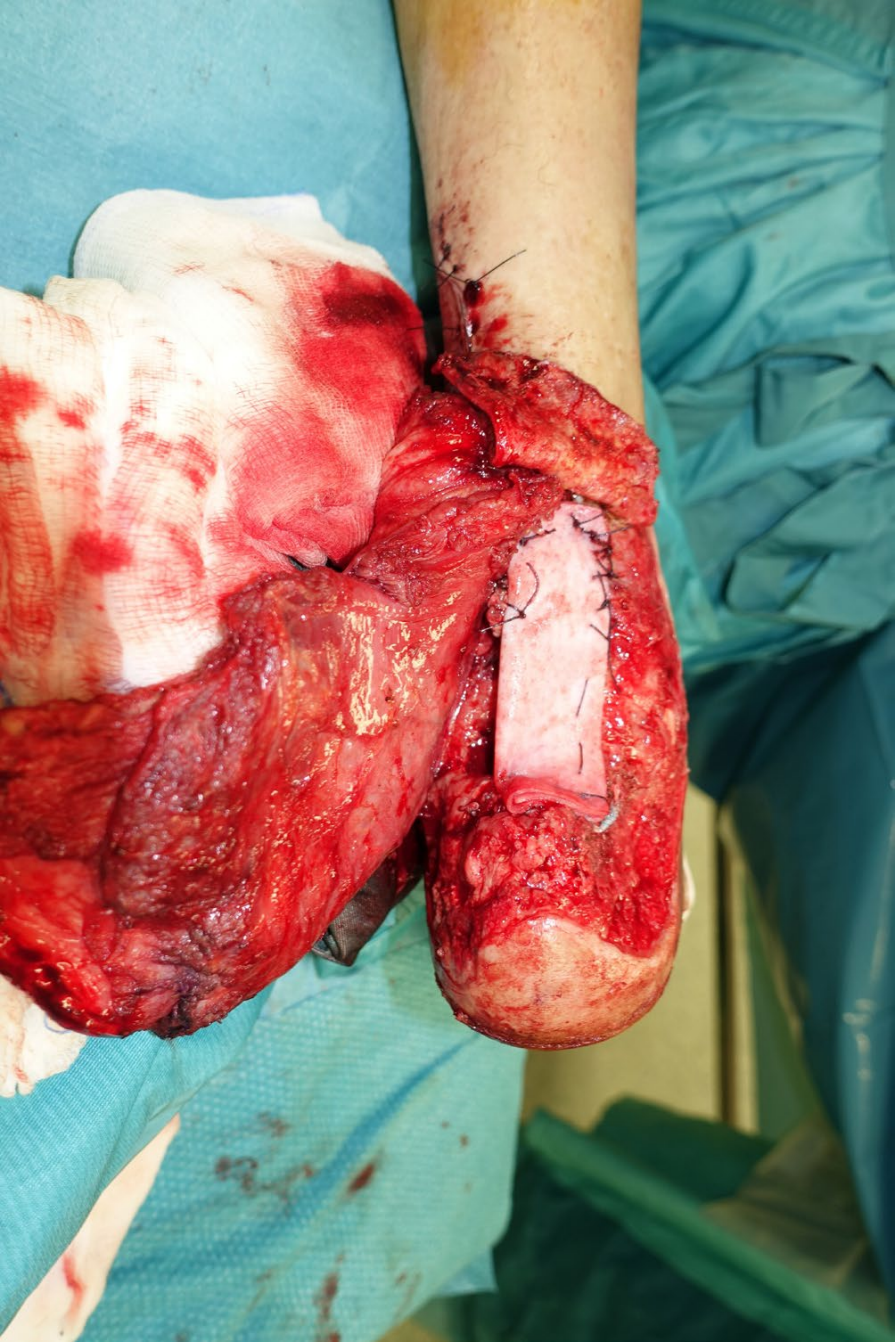
Hybrid Achilles tendon reconstruction with a 6‐cm‐long ADM neo‐tendon covered with a free myo‐cutaneous LD flap.

**FIGURE 4:**
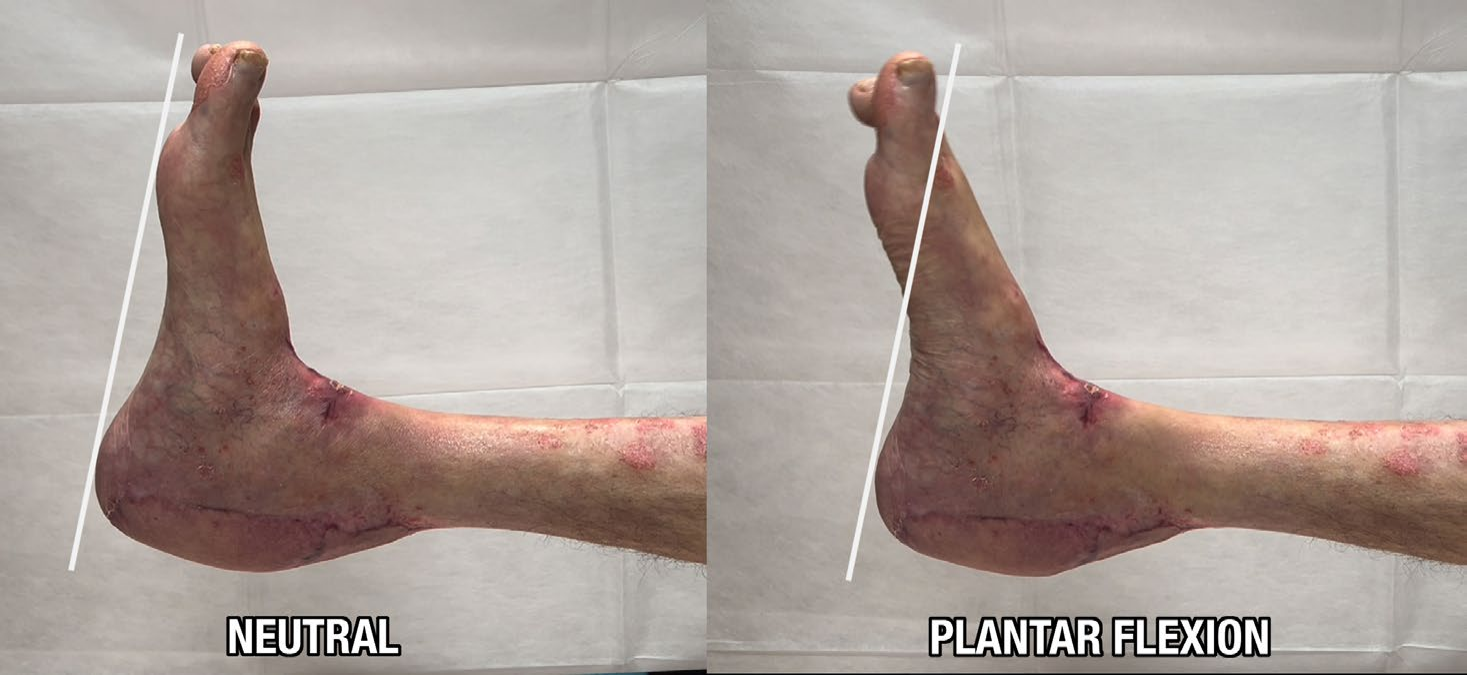
Clinical examinations 6 months postoperatively, with complete restoration of contour and function of the operated leg, with good excursion and complete integration of the reconstructed tendon.

The neo‐tendon was then wrapped in both anterior and posterior aspects with a free myo‐cutaneous LD flap with 16 × 10 cm skin paddle harvested from the contralateral side, the only reconstructive option spared by skin psoriasis (Figure 3), in order to maximize vascular ingrowth and guarantee functional excursion. Microvascular anastomoses were performed connecting the thoracodorsal pedicle to the posterior tibial pedicle in end‐to‐side fashion for the artery and end‐to‐end fashion for the vein. The part of the LD flap exceeding its skin paddle was resurfaced with split‐thickness skin grafts. The postoperative course was uneventful. A splint was put in place for 6 weeks. Load bearing on the affected limb was prohibited for 6 weeks postoperatively, after which the patient gradually started walking again thanks to a physiotherapy rehabilitation program of 2 months (Galluccio et al. 2024). The recovery of the patient was documented both clinically (Figure 4) and radiologically with MRI examination (Figure 2b) acquired at 6 months postoperative follow‐up: Complete restoration of contour and function of the operated leg, with good excursion and complete integration of the reconstructed tendon was observed, with the patient being able to wear normal shoes. Eight months postoperatively, the patient underwent a gait analysis instrumental evaluation at the Movement Analysis Laboratory of the Fondazione Don Carlo Gnocchi in Rome (Davis et al. 1991). The exam confirmed that the patient's gait was almost symmetric, as indicated by the very similar values for all the spatial–temporal parameters measured on the operated and contralateral sides. In conclusion, despite slight alterations in walking speed, and the decrease in range of motion and power at the ankle joint compatible with postoperative fibrosis, the patient recovered well through a physiological pattern of walking that was substantially symmetrical and with an adequate execution of all phases of the gait (Video S1).

### 2.2 Case 2

An 82‐year‐old patient with multiple comorbidities was referred to our sarcoma surgery department for a recurrent fibroblastic sarcoma arising in the lower third of the left leg. After neo‐adjuvant radiotherapy, the patient was then scheduled for wide excision and free flap reconstruction. The preoperative Angio‐CT showed complete occlusion of both anterior and posterior tibial arteries 12 cm below the knee, with the leg surviving only on the peroneal artery. After resection, a compound 17 × 14 cm defect of the medial lower third of the leg including an 8‐cm‐long full‐thickness defect of the Achilles tendon was observed. Immediate reconstruction was provided with a free chimeric ALT‐fascia lata flap anastomosed to the dorsalis pedis vessels, still patent in a retrograde flow fashion. The postoperative course was uneventful until the fifth postoperative day, when the flap was later found ischemic and not salvageable. A second reconstruction was planned in two stages: first creating a 15‐cm great saphenous vein AV loop anastomosed to the posterior tibial vessels 12 cm below the knee. One week later, the loop was re‐explored and its patency confirmed. Therefore, a contralateral free myo‐cutaneous LD flap with a 20 × 8 cm skin paddle was harvested and anastomosed to the divided loop. Achilles tendon continuity was restored by the use of a 10‐cm‐long XCM BIOLOGIC Tissue Matrix (DePuy Synthes, Johnson & Johnson) folded in a strong, three‐layered tridimensional structure, as mentioned above, and inserted distally with anchor screws at the level of the calcaneal bone and then sutured to the Achilles tendon proximal stump or to the remnants of the calf effectors with Prolene 0 under maximal tension (dorsal flexion of the foot). The neo‐tendon was then wrapped in both anterior and posterior aspects with the LD flap. Soft tissues were resurfaced with the flap skin paddle and split‐thickness skin grafts. The postoperative course was uneventful. The patient was discharged 4 weeks later and transferred to a physiotherapy center where complete rehabilitation was achieved according to the program described above. Eight months postoperatively, the patient was free from local recurrence (Video S1). The clinical and radiological examinations showed complete restoration of contour and function of the operated leg and good excursion and complete integration of the reconstructed tendon, with the patient fully able to walk without assistance or devices, being able to wear normal shoes. Unfortunately, the patient refused to be followed at the same rehabilitation center and was lost at follow‐up making it impossible to evaluate his ambulatory progress with gait analysis instrumental evaluation. Nevertheless, 15 months postoperative clinical functional evaluation was obtained showing a good degree of function (Video S1).

## 3 Discussion

Cole et al. (2018) and Bertasi et al. (2017) have already described the use of ADMs such as AF‐ADM (LifeNet Health) and M‐ADM (DermACELL, LifeNet Health) as augmentation repair for the Achilles tendon. Although such results proved to be certainly encouraging, in their series the ADM was used to augment and reinforce the native Achilles tendon, which was still in place unviolated. On the other hand, no report exists in the literature about the reconstruction of full‐thickness Achilles tendon defects using ADMs to completely replace the continuity of the tendinous structure, this topic being the real clinical innovation provided by our preliminary report.

The XCM matrix (Brunetti et al. 2011) consists of a porcine protein scaffold devoid of any cellularity to avoid rejection. We hypothesized that the initially inert matrix only acted as a temporary scaffold to attract and stimulate cell growth that quickly took its place in providing a neo‐tendon consisting of the patient's own fibrous tissues. In our cases, this process was obviously facilitated by the close contact of the graft with the highly vascularized tridimensional structure of the free flap, which was necessary to wrap around the neo‐tendon and provide its vascular ingrowth.

In our view, the advantages of using the hybrid free flap/ADM approach are several. In our series, ADM showed to be a reliable alternative to completely replace the Achilles tendon, proving to be expendable with all the possible flap solutions. In this scenario, we strongly believe that ADMs can be either used in combination with composite free flaps such as the chimeric ALT‐fascia lata flap or the teno‐cutaneous RF flap, with the purpose of reinforcing the tendinous structure inside the flap, or together with soft tissue–only flaps, as shown in our series (Brunetti et al. 2022). On the other hand, we admit that such a hybrid reconstruction, when compared to a totally autologous approach, carries the risk of ADM exposure and infection. For this reason, it should be carefully planned in order to maximize the surface contact with the soft tissue free flap. In this scenario, regardless of the flap composition, the microvascular transplantation allows to bring healthy tissue and good blood flow helping in washing out any harmful bacteria and instead delivering antibiotics to the infection site, reducing the risk of ADM infection which would render an explantation mandatory.

Our report inevitably comes with some limitations: patient population and the retrospective nature of the analysis. A wider, prospective, randomized, controlled trial study to compare the hybrid flap/ADM approach to other reconstructive options in use nowadays is certainly needed before we can draw any conclusions on its results and usefulness. Still, the hybrid approach, combining ADM to reconstruct Achilles tendon and free flap to provide neo‐tendon integration and soft tissue replacement, may be a good alternative and innovative solution for the reconstruction of complex Achilles tendon defects. Functional results, showing both patients now back to walking on their own, are more than promising for future studies on large series of patients.

## Author Contributions

All authors contributed to the conception and design of the study, data collection, analysis, and interpretation. Drafting and critical revision of the manuscript were carried out collaboratively by all authors. All authors approved the final version of the manuscript and agreed to be accountable for all aspects of the work.

## Conflicts of Interest

The authors declare no conflicts of interest.

## Supporting information


**Video S1:** Detailed surgical technique and outcomes of the two patients.

## Data Availability

All relevant data supporting the findings of this case report are contained within the article and its Supporting Information.
